# Chemical defense in developmental stages and adult of the sea star *Echinaster* (*Othilia*) *brasiliensis*

**DOI:** 10.7717/peerj.11503

**Published:** 2021-06-18

**Authors:** Renato Crespo Pereira, Daniela Bueno Sudatti, Thaise S.G. Moreira, Carlos Renato R. Ventura

**Affiliations:** 1 Department of Marine Biology, Universidade Federal Fluminense, Niterói, Rio de Janeiro, Brazil; 2 Invertebrate Department, Universidade Federal do Rio de Janeiro, Rio de Janeiro, Rio de Janeiro, Brazil

**Keywords:** Marine chemical ecology, Tropical region, Echinoderm, Starfish, Sympatric, Allopatric, Echinodermata, Feeding ecology, Anti-predation defense, Predation

## Abstract

To date, evidence regarding the performance of secondary metabolites from larval stages of sea stars as an anti-predation defense relates only to a few species/specimens from a few geographic ranges. Unfortunately, this hinders a comprehensive global understanding of this inter-specific predator-prey interaction. Here, we present laboratory experimental evidence of chemical defense action in the early developmental stages and adults of the sea star *Echinaster* (*Othilia*) *brasiliensis* from Brazil against sympatric and allopatric invertebrate consumers. Blastulae, early and late brachiolarias of *E*. (*O*.) *brasiliensis* were not consumed by the sympatric and allopatric crabs *Mithraculus forceps*. Blastulae were also avoided by the sympatric and allopatric individuals of the anemone *Anemonia sargassensis*, but not the larval stages. Extracts from embryos (blastula) and brachiolarias of *E*. (*O*.) *brasiliensis* from one sampled population (João Fernandes beach) significantly inhibited the consumption by sympatric *M. forceps*, but not by allopatric crabs and *A. sargassensi* anemone. In this same site, extracts from adults *E*. (*O*.) *brasiliensis* significantly inhibited the consumption by sympatric and allopatric specimens of the crab in a range of concentrations. Whereas equivalent extract concentrations of *E*. (*O*.) *brasiliensis *from other population (Itaipu beach)inhibited the predation by allopatric *M. forceps*, while sympatric individuals of this crab avoided the only the higher level tested. Then, early stages and adult specimens of *E*. (*O*.) *brasiliensis* can be chemically defended against consumers, but this action is quite variable, depending on the type (anemone or crab) and the origin of the consumer (sympatric or allopatric).

## Introduction

Sea stars are common and conspicuous components of benthic marine ecosystems. They are well-adapted to bottoms and hard substrates, and varied habitat-types, from intertidal to abyssal and from polar to tropical regions (Lawrence, 2013; Motti et al., 2018). Of singular scientific interest and relevant ecological prominence is its ability to regulate intertidal ecosystems (keystone species) along the Pacific coast of Canada and the United States, and also in Chile and New Zealand (see reviews in Menge (1982) and Menge & Sanford (2013)). Sea stars have attracted considerable scientific interest as fascinating sources of structurally diversified secondary metabolites (mainly saponins) exhibiting different potentials of pharmacological activities (Verbist, 1993; Dong et al., 2011) and ecological roles (McClintock, Amsler & Baker, 2013).

Sea stars have a remarkable chemosensory capacity. The secondary metabolites of sea stars have relevant ecological roles as chemical signals or cues for foraging, aggregation, reproduction, spawning and larval settlement (Motti et al., 2018). In addition, their secondary metabolites can provide chemical defense against potential predators. However, they can not escape very quickly from some predators, such as fishes and crabs (Amaral et al., 2008), anemones (Bos, Gumanao & Salac, 2008), polychaetes (Glynn, 1984), shrimps (Prakash & Kumar, 2013), birds (Wootton, 1997) and even other species of sea stars (McClintock et al., 2008). In the defensive context against consumers or potential predation, the diverse and sophisticated arsenal of secondary metabolites constitute a primary chemical defense system used by adults, eggs, embryos, larvae, and juveniles of sea stars (McClintock, Amsler & Baker, 2013). Therefore, the chemical substances produced by sea stars and their chemical perception of the environment allow these animals to present essential strategies of survival in different habitats.

Dramatic escape reaction of marine invertebrates to continually secreted steroid by adult sea stars was one of the first signals of defensive action of secondary metabolites in these organisms (Mackie, Lasker & Grant, 1968; Mackie, 1970; Feder, 1972). Several studies evidenced the chemical defense property of these chemicals in sea stars through of the detection of low susceptibility of live organisms, fresh body-wall and artificial food pellets containing extracts of several species to consumption by fish and spider crab (Bryan, McClintock & Hopkins, 1997; Bryan, Rittschof & McClintock, 1996; McClintock et al., 2006). Besides, extracts from sea stars promote several behavioral responses as an expression of deleterious effects, such as ichthyotoxicity effects (*e.g.*, behavioral changes and mortality) in fishes (Mackie, Singh & Fletcher, 1975; McClintock, 1989), inhibition of the consumption by another sea star (Taboada, Núñez-Pons & Avila, 2013) and promotion of swimming response in anemone (Pathirana & Andersen, 1986).

Like the adults, organs and the early stages of sea star (ovaries, eggs, embryos, and juveniles) also exhibit chemical defense capable of inhibiting consumption by marine fishes (Lucas, Hart & Salathe, 1979; McClintock & Vernon, 1990) and invertebrates, such as amphipod, isopod, anemone and sea star (McClintock & Baker, 1997; Iyengar & Harvell, 2001; McClintock et al., 2003). Presumably, the initial developmental stages of sea stars have the same active chemical components used as a chemical antipredation defense in parental adults, as observed in eggs and larvae of *Acanthaster planci* (Lucas, Hart & Salathe, 1979).

However, the chemical defenses of sea stars are not efficient for all potential predators. Therefore, broad approaches to investigate the action of the antipredation chemical protection is still necessary, since secondary metabolites may exhibit multiple other ecological roles besides defensive property against consumers. For example, the polychaete *Pherecardia striata* can chemically perceive injured *Acanthaster planci* (and other prey) by weak directional signals (Glynn, 1984). The Giant Triton snail, *Charonia tritonis*, detects *Acanthaster planci* odor plume to locate its prey (Hall et al., 2017).

Contents and compound-types of defensive secondary metabolites in marine organisms may occur at several distinct levels, such as among populations from different habitats (*e.g.*, De Caralt et al., 2013). The knowledge of effective defensive chemical action of sea star larva against predators is still of restricting spatial scale since investigations focused mainly on Antarctic sea star species (*e.g.*, McClintock, Amsler & Baker, 2010, 2013). There is little other evidence from species in the United States, San Juan Island, Washington (Iyengar & Harvell, 2001), and North Carolina (Bullard, Lindquist & Hay, 1999). Efforts to increase studies in other regions of the world will provide a global outlook of the occurrence of antipredation chemical defense in sea stars, especially in tropical areas where selective pressure due to predation is supposed more intense (Freestone et al., 2011). A worldwide overview can help in understanding the magnitude and evolutionary aspects related to chemical protection in sea stars. Besides that, information about chemical defenses at different stages in the life cycle of starfish does not reveal a clear pattern. Rare are the studies that used live larvae as well as the extract from the different stages of the life cycle, thus isolating the presence of defensive metabolites and the palatability of the larva inherent to the nutritional value (McClintock, Amsler & Baker, 2013).

This study aims to verify whether adults and initial stages of development (embryos on blastula and brachiolaria stages) of the sea star *Echinaster* (*O*.) *brasiliensis* contain chemical defense that may deter predation in sympatric and allopatric predators. This starfish is a suitable model for this experimental study because we have already known much of its biology in the field, like its reproductive cycle, early development, and feeding. We also know how to maintain it in the laboratory (Lopes & Ventura, 2016).

## Methods

### Collect of adult sea stars, anemones, and crabs

Adult specimens of *Echinaster* (*O*.) *brasiliensis* (5.6 cm ± 0.67) and their potential consumers assayed, the crab *Mithraculus forceps* (1.0 cm ± 0.24) and the anemone *Anemonia sargassensis* (1.5 cm ± 0.21) were collected by snorkeling from two beaches on the coast of the Rio de Janeiro State: João Fernandes (JF), at Armação dos Búzios city (22°44′30″ S; 41°52′31″ W) and Itaipu (IT), at Niterói city (22°58′22″ S; 43°02′44″ W). Crabs are predators of starfish (e.g. Pratchett, Vytopil & Parks, 2000) and filter-feeding anemones are used as a chemical defense detection model in stages of development of marine invertebrates (see Lindquist, Hay & Fenical, 1992; Lindquist & Hay, 1996). Thus, due to the lack of information on *E*. (*O*.) *brasiliensis* consumption and its stages of involvement by these consumers, *M. forceps* and *A. sargassensis* are potential predator.

In the laboratory, individuals of sea stars, crabs and anemones were kept in individualized aquariums of 5L, 250 and 250 ml, respectively, at a temperature of 18 (±2), salinity around 35 PSU and natural seawater maintained in glass bottles before introduction in a recirculating and filtered system.

The sea stars fed on biofilm accumulated on the wall of the aquariums (personal observation) and, probably, on organic matter presumably dissolved in the water, as already known in the literature (Ferguson, 1969). They were used to obtain the different life cycle stages that were used in the bioassays with organisms and their extracts.

The anemones and crabs were pre-conditioned to eat the commercial feeds Botton Fish, Alcon Co. and the lyophilized *Gammarus* sp., respectively, for three consecutive days through a Pasteur pipette as a way of “training” them to consume in a similar manner further used in the bioassay. Considering the lack of knowledge regarding the nutritional value of embryos and larvae of the species *E*. (*O*.) *brasiliensis*, we opted for the use of a ration that presented a nutritional value similar to that of the gonads of individuals belonging to the genus *Echinaster* (Scheibling & Lawrence, 1982). For adults, the caloric value of the species itself was used (according to Diniz et al., 2014). However, the grains of Botton Fish were fractionated into small pieces to simulate the same size of an *E*. (*O*.) *brasiliensis* embryo or larva. The same procedure was done with the *Gammarus* sp., whose only part of the head of the amphipod was used.

### Obtaining of gametes, embryos, and larvae of *E*. (*O*.) *brasiliensis*

After one week maintained under laboratory conditions, four to five individuals of *E*. (*O*.) *brasiliensis* from JF were replaced by each other until spawning occurred. Sea stars spawned spontaneously without the need for external inducers, that is, the combination of at least one male specimen and a female. Although it is impossible to assure which individuals took part in fertilization, more than two specimens produced the offspring each time. Considering that more than a couple of adults spawned and we had different spawn events, we can expect some genetic diversity among offspring. After certifying the occurrence of fertilization under a stereoscopic microscope, fertilized eggs were maintained in aquaria with seawater. Three distinct stages (defined according to Lopes & Ventura (2016)) were obtained and used in the bioassays: blastulae (on 1–2 days post fertilization), early and late brachiolaria larvae (3 and 6–13 days post fertilization, respectively).

### Obtaining of extracts from embryos, larvae and adults of *E. brasiliensis*

**Figure 1:**
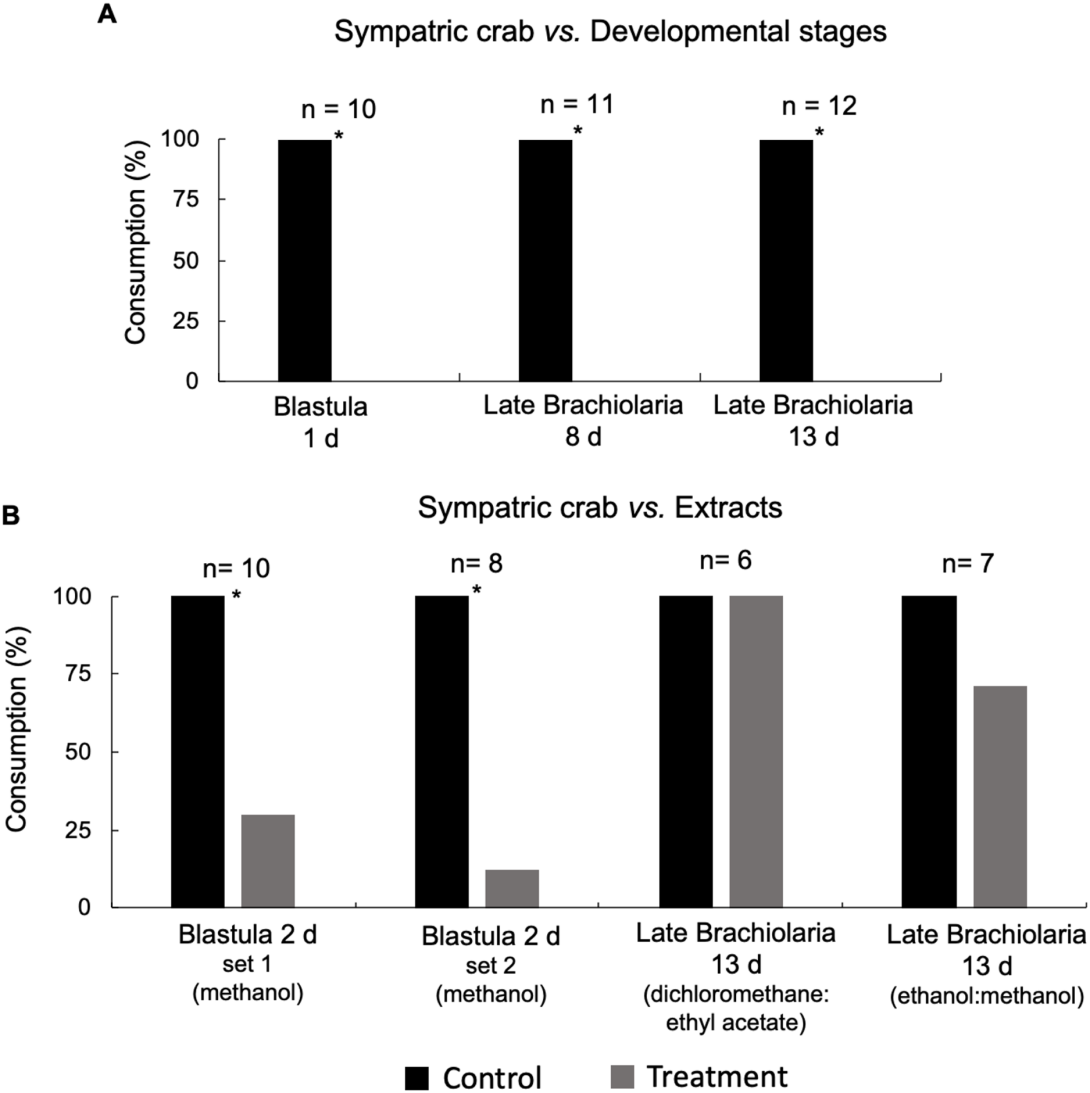
(A) Susceptibility of blastula (1 day) and late brachiolaria larvae (8 and 13 days) stagesof *E. (O.) brasiliensis* from João Fernandes beach to consumption by sympatric crab *M. forceps*. (B) Defensive effect of extracts from blastula (2 days—methanol), late brachiolaria larvae (13 days—dichloromethane:ethyl acetate—80:20) and (ethanol:methanol—80:20) of *E. (O.) brasiliensis* from João Fernandes against sympatric crab *M. forceps*. * Significant difference, Fisher test, *P* < 0.05.

**Figure 2:**
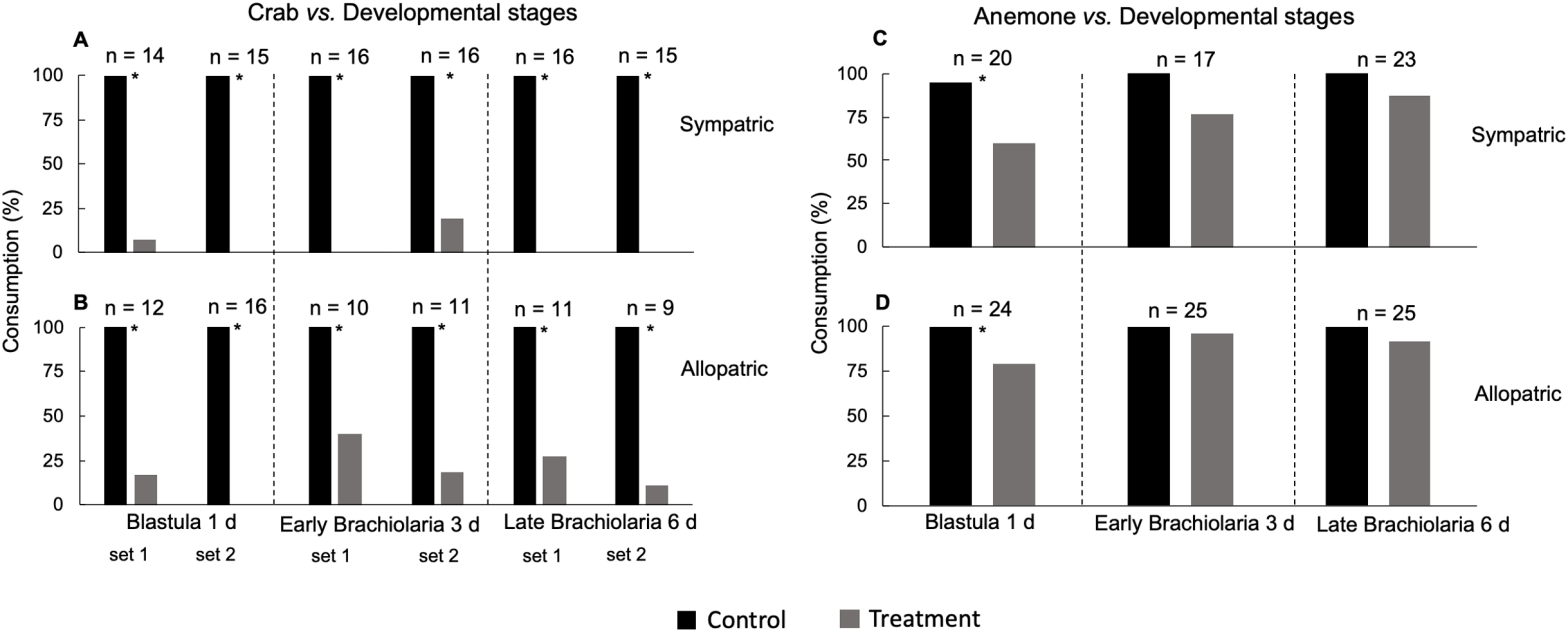
Susceptibility of blastula (1 day), early (3 days) and late (6 days) brachiolaria stages of *E*. (*O*.) *brasiliensis* from João Fernandes beach to consumption by sympatric (A) and allopatric (B) crab *M. forceps;* sympatric (C) and allopatric (D) sea anemone *A. sargassensis*. *Significant difference, Fisher test, *P* < 0.05. Bioassay replicate are indicated as set 1 and set 2.

**Figure 3:**
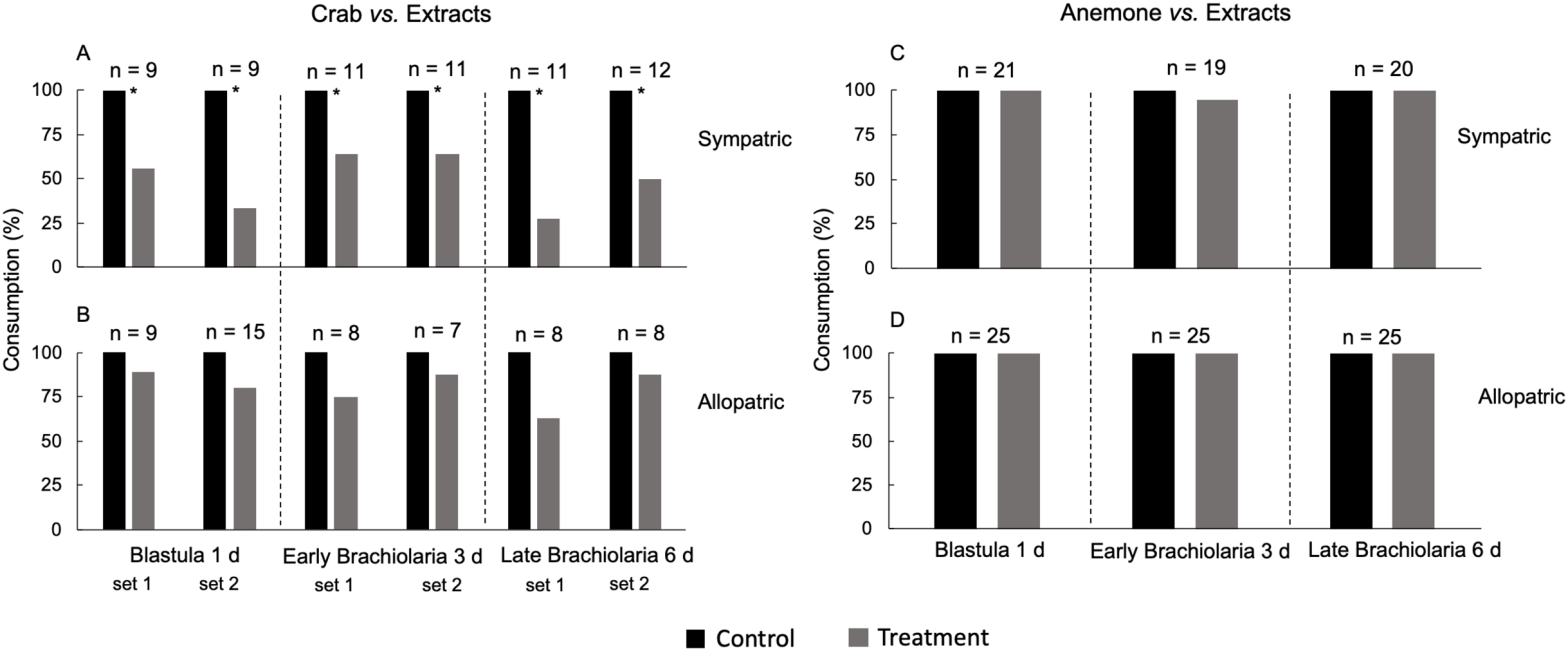
Defensive effect of methanolic extracts (natural concentration) from blastula (1 day), early (3 days) and late brachiolaria (6 days) of *E*. (*O*.) *brasiliensis* from João Fernandes beach against sympatric (A) and allopatric (B) crab *M. forceps;* sympatric (C) and allopatric (D) sea anemone *A. sargassensis*. *Significant difference, Fisher test, *P* < 0.05. Bioassay replicate are indicated as set 1 and set 2.

**Figure 4:**
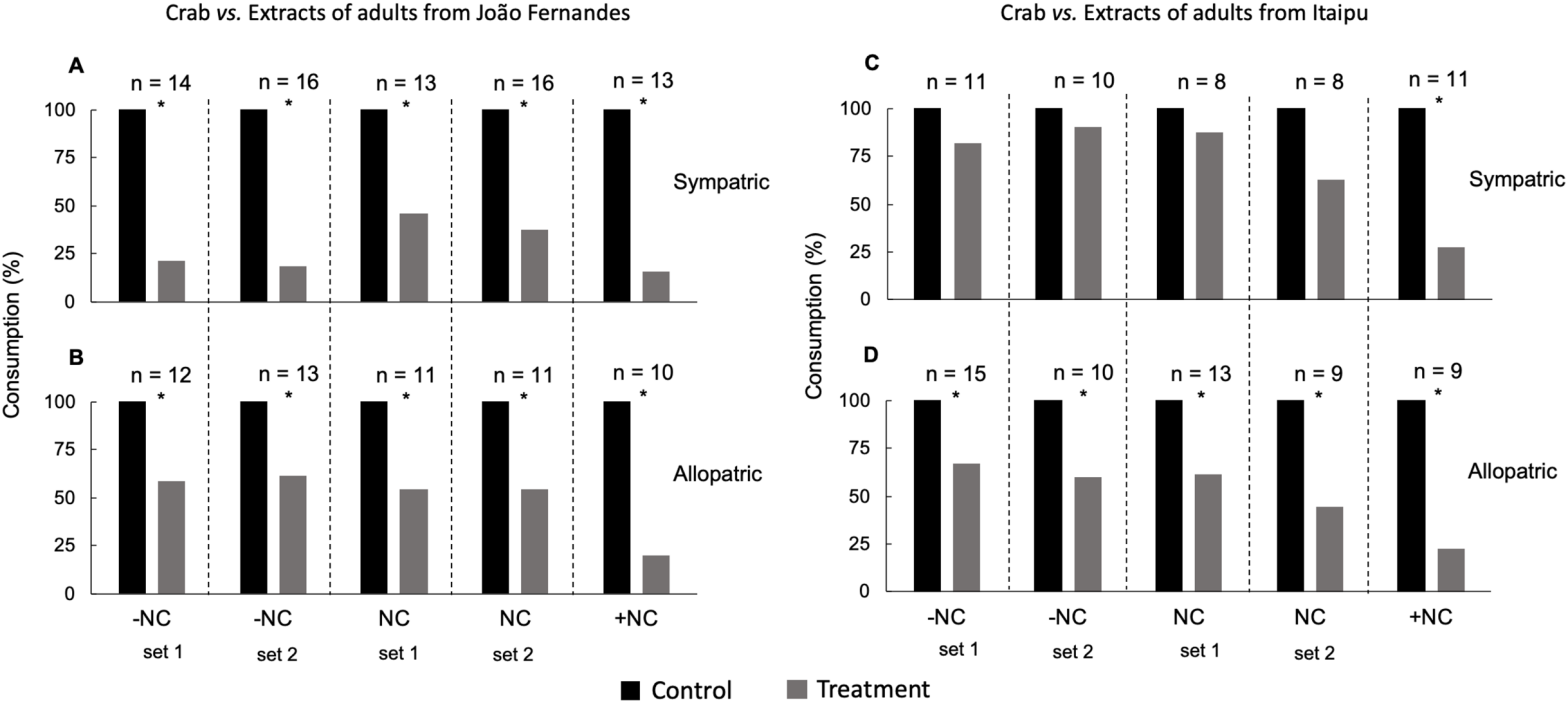
Defensive effect of different concentrations of extracts from adults of *E*. (*O*.) *brasiliensis* from JF against the sympatric (A) and allopatric (B) crab *M. forceps*; and from Itaipu against (C) sympatric and allopatric (D) crab *M. forceps*. −NC = 2.5-fold below natural concentration; NC = natural concentration; +NC = 4-fold above natural concentration. *Significant difference, Fisher test, *P* < 0.05. Bioassay replicate are indicated as set 1 and set 2.

Blastula (2 days) and late brachiolaria (13 days) larvae were extracted with organic solvents to explore a broad polarity range: dichloromethane:ethyl acetate (80:20), ethanol:methanol (80:20) and methanol (100%) and to be used in the first bioassays carried out in order to define which solvent would be the most appropriate to extract chemicals exhibiting defensive property in *E*. (*O*.) *brasiliensis* (Figs. 1A and 1B in the results). Once the methanolic extract was found to be the most effective, it was used in all remaining bioassays performed in this study (Figs. 2–4 in the results).

Before extraction, embryos and larvae were carefully dried using a syringe and absorbent paper (for 20 s) to remove excess water, then weighed on a precision scale (Sartorius Mark, Model CP225D). Subsequently, known mass of each early developmental stages (embryos and larvae and using the same mass/solvent volume ratio) were extracted separately by immersion in the solvent left to steep in a drying hood for 48hr. Fresh solvent was added and the process repeated a further two times.

Adult specimens of *E*. (*O*.) *brasiliensis* from João Fernandes (JF) and Itaipu (IT) beaches were cleaned of sediment by simple washing in sea water, wet weighed (Sartorius Mark, Model CP225D), and then frozen. Frozen specimens were broken into pieces, lyophilized, and reweighed after which they were extracted in 100% methanol (100 mL), with solvent changes occurring three times every 48hr (as described above). Although the metabolites are on the wall body of the sea stars (Demeyer et al., 2014), we opted for the extraction of the entire body due to the small number of individuals we obtained.

Extracts from developmental stages and adults were filtered (Schleicher & Schuell Mark, 12.5 cm day. Filtration membranes), and dried using a rotary evaporator (Büchi R-114, Bath B-480 coupled to the vacuum pump Thomas Scientific Pump DOA-P104E-AA).

### Bioassays general procedures

Food preference bioassays were conducted whereby artificial foods which were alternately offered to predators. Predators had to choose control or treatment food (containing extracts from adults of *E*. (*O*.) *brasiliensis* or from the early developmental stages – embryos and larvae; see Lindquist, Hay & Fenical (1992)). The images recorded during the tests were made available in the (Videos S1–S4). As the nutritional value and mode of application can influence the decision to choose the food item (Pawlik, 2012), all predators were pre-conditioned and the tests started only after the confirmation that the consumers accepted the artificial foods.

### Susceptibility of *E. (O.) brasiliensis* embryos and larvae to consumption

As this is the first study on chemical defense in *E. (O.) brasiliensis*, some initial tests were carried out in order to know the susceptibility of some developmental stages of this sea star to consumers. The susceptibilities of blastula (2 days) and late brachiolaria (8 and 13 days) of *E*. (*O*.) *brasiliensis* were evaluated to consumption by the crab *M. forceps*. Further, several other feeding assays were carried out with to obtain a broad characterization about the susceptibility of some developmental stages of *E*. (*O*.) *brasiliensis* by to consumption by sympatric and allopatric specimens of the crab *M. forceps* and the anemone *A. sargassensis*.

For all assays, treatments (embryos or larvae) and controls (fish ration and lyophilized *Gammarus* sp., for crabs and anemones, respectively) were alternately offered to individual consumers in separate aquaria to verify their acceptance or rejection, *i.e*., whether the predator consumed the food item offered or not.

A variable number of replicates was used in trials with sympatric (*n* = 10–16) and allopatric (*n* = 9–16) specimens of the crab *M. forceps* and stages of *E*. (*O*.) *brasiliensis*, and sympatric (*n* = 17–23) and allopatric (*n* = 24–25) specimens of anemones *A. sargassensis*. When bioassays with embryos and larvae were conducted in replicate, they were indicated as set 1 and set 2. The results are presented as a percentage of consumption, which corresponds to the number of food items consumed or not.

### Defensive property of extracts from embryos, larvae and adults of *E. (O.) brasiliensis*

In the first assays, extracts obtained with methanol, dichloromethane:methanol and ethanol:methanol from blastula (2 days) and late brachiolaria (13 days) developmental stages of *E*. (*O*.) *brasiliensis* and respective controls were alternately offered to *M. forceps* individuals to verify the defensive action against this crab. These assays were carried out to verify the existence of defensive property in chemicals from of *E*. (*O*.) *brasiliensis* and what would be the better solvent to express this property. After verify that methanol was the better solvent when compared with the combination dichloromethane:methanol and ethanol:methanol, it was used all the remaining extractions and bioassays. Methanol is used for saponin extraction, since this is the most abundant class of secondary metabolites found in sea stars (*e.g.*, De Marino et al., 1998). These chemicals were incorporated into artificial foods, the commercial Botton fish, Alcon Co. and the lyophilized *Gammarus* sp., for use in anemone and crab bioassays, respectively.

The same amount of solvent was added to control food in an equivalent mass used in the extraction. After the complete evaporation of the solvent, control and treatment foods were offered to predators. The defensive action of the extracts was evaluated based on their natural concentrations from each developmental stages and adults of *E*. (*O*.) *brasiliensis*, represented by the ratio between the dry weight of the extract and the wet weight of the live specimens extracted. However, given the issue about imprecisions of estimation the natural concentration in adult specimens, a range of broad concentrations was chosen for tests: natural concentration (NC), 2.5-fold below natural concentration (−NC) and 4.0-fold above natural concentration (+NC).

A variable number of specimens was used in trials with extracts from embryos and larvae against sympatric (*n* = 6–12) and allopatric crabs (*n* = 7–16), also for sympatric (*n* = 19–21) and allopatric anemones (*n* = 20–25). Such variations also occurred in bioassays with the extract from adults of *E*. (*O*.) *brasiliensis*, comprising sympatric (*n* = 13–16) and allopatric (*n* = 10–13) crabs from João Fernandes beach; and sympatric (*n* = 8–11) and allopatric (*n* = 9–15) crabs from Itaipu.

Artificial food and respective control were alternately offered to consumers, *M. forceps* and *A. sargassensis*. For bioassays with anemones, each food item (control and treatment) was offered alternately to them up to a maximum of three attempts via a Pasteur pipette for confirmation of the choice by one of them. If there was first-time acceptance (Video S1), no new offers were made. But if the food item was rejected (Video S2), it was offered a second time or a third time after rejection. Similarly, food items (control, Video S3 and treatments, Video S4) were also alternately offered to crabs, but with offers made up to five times until their option for a food item, since crabs are very active and sometimes moved away but did not consume any of the food items offered to them.

### Data analysis

Significant differences related to acceptance or rejection of controls and treatments were determined by Fisher’s non-parametric test (one-tailed test), assuming α = 5% and using the Statistics 8.0 software. The null hypothesis (H_0_) for this test is that the foods offered (control and treatment) would be consumed in the same proportion and that in the alternative hypothesis (H_1_), treatment would be rejected in greater proportion.

## Results

The sympatric crab *M. forceps* did not consume initial stages of development of *E*. (*O*.) *brasiliensis* (blastula, 1 day: *p* < 0.001; 8 days larvae: *p* < 0.001; 13 days larvae: *p* < 0.001) (Fisher test, Fig. 1A). In addition, metanolic extract from blastulae inhibited the consumption by the sympatric crab *M. forceps* (Fisher test, *P* < 0.05; Fig. 1B), while extracts of other polarities (dichloromethane:ethyl acetate and ethanol:methanol) obtained from the brachiolaria stages exhibited no inhibitory effect (Fisher test, *P* > 0.05; Fig. 1B). Due this clear evidence that the methanol extract expressed defensive action, this solvent was used in the remaining experiments of this study.

Stages of blastula and early brachiolaria larvae (3 and 6 days) was rejected by sympatric (Fig. 2A) and allopatric (Fig. 2B) crabs *M. forceps* (Fisher test, *P* < 0.05), whereas sympatric and allopatric anemone *A. sargassensis* rejected the blastula stage (1 day), but consumed early brachiolaria stages (3 and 6 days) (Fisher exact test, *P* < 0.05; Figs. 2C and 2D).

Natural concentrations of methanolic extracts from blastulae (1 day) and early and late brachiolarias (3 and 6 days, respectively) of *E*. (*O*.) *brasiliensis* inhibited the consumption by sympatric *M. forceps*, but not for allopatric crab specimens (Fisher exact test, *P* < 0.05; Figs. 3A and 3B). However, extracts of these three developmental stages were palatable to both sympatric and allopatric *A. sargassensis* (Fisher exact test, *P* > 0.05; Figs. 3C and 3D).

Consumption by sympatric and allopatric individuals of the crab *M. forceps* was inhibited by different concentrations of extracts (−NC, NC, +NC) from adults of *E*. (*O*.) *brasiliensis* from João Fernandes (JF) specimens (Fisher test, *P* < 0.05; Figs. 4A and 4B). Sympatric crabs only rejected the highest concentration (+NC) extracts from IT specimens of *E*. (*O*.) *brasiliensis* (Fisher exact test, *P* < 0.05; Fig. 4C) and they were observed consuming those at the natural and lower than natural concentrations (Fisher exact test, *P* > 0.05; Fig. 4C), while allopatric specimens of this crab avoided all concentrations of these extracts (Fisher exact test, *P* > 0.05; Fig. 4D). The complete results of Fisher’s non-parametric tests can be accessed in the (Table S1).

## Discussion

Seventy-seven species of sea stars are found on the Brazilian coastline (Ventura et al., 2013). Ventura et al. (2013) reported the historical records and current research of echinoderms in Brazil. Although substantial improvement in scientific knowledge on echinoderms from Brazil has been made, the lack of studies on the chemical ecology of sea stars is evident. Here, we show evidence that different developmental stages of the sea star *E*. (*O*.) *brasiliensis* including blastulae (planktonic) and early (planktonic) and late brachiolaria larvae (undergoing settlement) exhibit chemical defense, which can vary with predator identity and condition of sympatry or allopatry.

For decades, some studies placed significant importance on the putative high predation pressure exerted over larvae of marine invertebrates to explain population density levels (Young & Chia, 1987; McClintock & Baker, 1997). However, several studies have since demonstrated the presence and action of chemical defenses in several marine invertebrates, such as gorgonians (Harvell, West & Griggs, 1996), hydroids, sponges, and bryozoan (Lindquist & Hay, 1996). It has also been revealed that different developmental stages of sea stars have a chemical defense arsenal capable of protecting them against potential consumers (Lucas, Hart & Salathe, 1979). Here we show the three developmental stages (blastula, and early and late brachiolaria) of the sea star *E*. (*O*.) *brasiliensis* are chemically defended against consumption by the crab *M. forceps*. In contrast, the early and late brachiolarias were susceptible to predation by anemone *A. sargassensis*. In general, anemones are considered potential predators of many invertebrate larvae (Young & Chia, 1987; Iyengar & Harvell, 2001). The distinct developmental stages of *E*. (*O*.) *brasiliensis* are differentially susceptible to predation and predator-type, with chemical defense playing a key role.

The methanolic extract was found to be the most active against potential predators when compared dichloromethane:ethyl acetate (80:20) and ethanol:methanol extracts (80:20), according with our first assays. Although the present study did not identify the compound(s) responsible for the defensive action observed, it is highly likely to be a saponin. Sea star-derived saponins are of the steroid-glycoside type (Minale, Riccio & Zollo, 1995), commonly known as asterosaponins (Dini et al., 1983). Both metabolites are abundant in sea stars and compatible with this solvent polarity (*e.g.*, De Marino et al., 1998).

Of all assayed developmental life-stages of *E*. (*O*.) *brasiliensis*, the blastulae were better chemically-defended against its two potential predators, the crab *M. forceps* and the anemone *A. sargassensis*. Furthermore, this defensive action of chemicals is broad-spectrum, as it inhibits the consumption by both sympatric and allopatric *M. forceps*.

In this first day of development, planktonic blastulae have no physical or behavioral defenses (Iyengar & Harvell, 2001), and as they do not have cilia and are unable to swim they tend to be more vulnerable to predation. However, the presence of secondary metabolites able to elicit a chemical defense in *E*. (*O*.) *brasiliensis* seems to compensate for these limitations. The cilia in larvae of *E*. (*O*.) *brasiliensis* only appear in the brachiolaria stage (Lopes & Ventura, 2016), and may constitute a physical defense. The same occurs in other echinoderms (Emlet, 1994) and other marine invertebrates (Chia, Buckland-Nicks & Young, 1984). This feature provides high dispersion capacity in the short time in which lecithotrophic larvae remain in the water column (Dyrynda, Pipet & Ratcliffe, 1995; Lindquist & Hay, 1996). As such, sea star larvae rely on the joint action of chemicals (even at lower concentrations), physical (cilia), and behavioral (in synergy or complementary roles) strategies to avoid or minimize predation.

A change from chemical to other structural defensive strategies may result in a decrease in the concentration of active chemical along the developmental stages. For example, the concentration of defensive compounds in chemically defended larvae of polychaetes declines throughout their lifespan (Cowart et al., 2000). Chemical defense levels in embryos and larvae of soft coral *Briareum asbestinum* also oscillate during ontogeny, drastically decreasing as they undergo developmental transitions to reach the juvenile stage (Harvell, West & Griggs, 1996). This support the concept that blastulae and larvae of *E*. (*O*.) *brasiliensis* would be chemically well-defended against predators but could employ other strategies in later stages of development. Furthermore, even if the content of the chemical defense decreases as development progresses, it is still sufficient in the brachiolaria to protect them from predation by *M. forceps*. The presence of defensive chemicals in the blastulae suggests a parental source, which decreases as they develop and transition to juveniles (Lucas et al., 1979). However, other authors argue that defensive substances do not come merely from the parents, but that the larvae themselves can produce these chemicals (Cowart et al., 2000; Iyengar & Harvell, 2001).

Yolky eggs and lecithotrophic larvae of echinoderm are more vulnerable to predation because they are visible and possess conspicuous in color (Bosch, 1989; Pearse, McClintock & Bosch, 1991), are incapable to swimming (Bosch, 1989; Pearse, McClintock & Bosch, 1991) and have high energy content (McClintock & Pearse, 1986), all characteristics that organisms that likely process feeding deterrents (Iyengar & Harvell, 2001), and also known for *E*. (*O*.) *brasiliensis* (Atwood, 1973; Lopes & Ventura, 2016). Chemically defended larvae are often conspicuous, being active during the day, and remaining close to the benthos during their short swimming stage - lecithotrophic larvae (Lindquist, 2002). Furthermore, both non-feeding (*e.g.*, soft corals—Harvell, West & Griggs, 1996; and ascidians—Lindquist, Hay & Fenical, 1992) and feeding (*e.g.*, sea stars—Lucas, Hart & Salathe, 1979; sea-urchins—Cowden, Young & Chia, 1984; and polychaetes—Cowart et al., 2000) larvae can reduce predation through the chemical defense. Therefore, there is still no consensus on whether the chemical defense would be related to larvae-type.

Since *E. (O.) brasiliensis* exhibits a widespread geographic distribution, extending from Florida to the Gulf of San Matias, in Argentina (Clark & Downey, 1992, Amaral et al., 2008; Lopes & Ventura, 2016), we can presuppose that the defensive action evidenced here may be a broad scale strategy for this sea-star species.

Adults from both localities, João Fernandes and Itaipu beaches inhibited sympatric and allopatric crabs. However, the extract from Itaipu specimens was only active in a concentration higher than the natural against sympatric crabs, which may indicate a local adaptation to chemical defense. As the secondary metabolites are in the body wall of the sea star (Demeyer et al., 2014), and we extract the entire body of *E. (O.) brasiliensis*, the natural concentration tested was below the natural one. Consumers that has had no previous contact with *E. (O.) brasiliensis* and their developmental stages would not have developed adaptations for resistance to levels of chemical defense produced by this sea star. Here, we use the sympatric and allopatric approaches to evaluate the defensive chemistry of *E. (O.) brasiliensis*, which allows us to assess the magnitude of the defensive action.

Although our findings are not novelty in the sea stars context, they have significance in the context of the geographical range of chemical defense production by marine organisms and also in a biogeographic approach. Previous data on chemical defense of sea star species are prolific and well-explored in studies carried out in Antarctic region (see McClintock, Amsler & Baker, 2010, 2013), and only few examples from other regions in USA regions, Washington (Iyengar & Harvell, 2001) and North Carolina (Bullard, Lindquist & Hay, 1999). But chemical defense in sea stars may be also important in tropical regions as evidenced in the present study for one species in South Atlantic.

## Supplemental Information

10.7717/peerj.11503/supp-1 Supplemental Information 1 Video: Consumption of control by anemone.

10.7717/peerj.11503/supp-2 Supplemental Information 2 Raw data for Figure 1.

10.7717/peerj.11503/supp-3 Supplemental Information 3 Video: Rejection of treatment by anemone.

10.7717/peerj.11503/supp-4 Supplemental Information 4 Raw data for Figure 2.

10.7717/peerj.11503/supp-5 Supplemental Information 5 Raw data for Figure 3.

10.7717/peerj.11503/supp-6 Supplemental Information 6 Video: Consumption of control by crab.

10.7717/peerj.11503/supp-7 Supplemental Information 7 Raw data for Figure 4.

10.7717/peerj.11503/supp-8 Supplemental Information 8 Video: Rejection of treatment by crab.

10.7717/peerj.11503/supp-9 Supplemental Information 9 Statistical tests. The complete results of Fisher’s non-parametric tests.

## Additional Information and Declarations

### Field Study Permissions

The following information was supplied relating to field study approvals (i.e., approving body and any reference numbers): Field experiments were approved by Instituto Chico Mendes de Conservação da Biodiversidade—ICMBio.

### Data Availability

The following information was supplied regarding data availability: The raw data are available in the Supplemental Files.
