# Supplementary material for: Chemical defense in developmental stages and adult of the sea star Echinaster (Othilia) brasiliensis
Source: PeerJ. 2021 Jun 18;9:e11503. doi: 10.7717/peerj.11503 (PMC8216172; doi:10.7717/peerj.11503)
Supplement: Supplemental Information 9 — The complete results of Fisher’s non-parametric tests. [file peerj-09-11503-s009.pdf]

Bioassay	Fisher's exact test			
**Sympatric crab *vs*. Developmental stages**	**n**	**df**	**p**	**Figure**
Blastula 1d	10	1	<0.0001	1A
Late Brachiolaria – 8d	11	1	<0.0001	1A
Late Brachiolaria – 13d	12	1	<0.0001	1A
Blastula 1d – set 1	14	1	<0.0001	2A
Blastula 1d – set 2	15	1	<0.0001	2A
Early Brachiolaria – 3d – set 1	16	1	<0.0001	2A
Early Brachiolaria – 3d – set 2	16	1	<0.0001	2A
Late Brachiolaria – 6d – set 1	16	1	<0.0001	2A
Late Brachiolaria – 6d – set 2	15	1	<0.0001	2A
**Allopatric crab *vs.* Developmental stages**				
Blastula 1d – set 1	12	1	<0.0001	2B
Blastula 1d – Set 2	16	1	<0.0001	2B
Early Brachiolaria – 3d – set 1	10	1	0.0054	2B
Early Brachiolaria – 3d – set 2	11	1	0.0001	2B
Late Brachiolaria – 6d – set 1	11	1	0.0005	2B
Late Brachiolaria – 6d – set 2	9	1	0.0002	2B
**Sympatric anemone *vs.* Developmental stages**				
Blastula 1d	20	1	0.0098	2C
Early Brachiolaria – 3d	17	1	0.0513	2C
Late Brachiolaria – 6d	23	1	0.1167	2C
**Allopatric anemone *vs*. Developmental stages**				
Blastula 1d	24	1	0.0248	2D
Early Brachiolaria – 3d	25	1	0.5000	2D
Late Brachiolaria – 6d	25	1	0.2449	2D
**Sympatric crab *vs*. extracts**				
Blastula 2d (methanol) – set 1	10	1	0.0015	1B
Blastula 2d (methanol) – set 2	8	1	0.0007	1B
Late Brachiolaria – 13d (dichloromethane:ethyl acetate)	6	1	1.0000	1B
Late Brachiolaria – 13d (etanol:methanol)	7	1	0.2308	1B
Blastula 1d – set 1	9	1	0.0412	3A
Blastula 1d – set 2	9	1	0.0045	3A
Early Brachiolaria – 3d – set 1	11	1	0.0451	3A
Early Brachiolaria – 3d – set 2	11	1	0.0451	3A
Late Brachiolaria – 6d – set 1	11	1	0.0005	3A
Late Brachiolaria – 6d – set 2	12	1	0.0069	3A
**Allopatric crab *vs*. extracts**				
Blastula 1d – set 1	9	1	0.5000	3B
Blastula 1d – set 2	15	1	0.1121	3B
Early Brachiolaria – 3d – set 1	8	1	0.2333	3B
Early Brachiolaria – 3d – set 2	7	1	0.5000	3B
Late Brachiolaria – 6d – set 1	8	1	0.1000	3B
Late Brachiolaria – 6d – set 2	8	1	0.5000	3B
**Sympatric anemone *vs*. extracts**				
Blastula 1d	21	1	1.0000	3C
Early Brachiolaria – 3d	19	1	0.5000	3C
Late Brachiolaria – 6d	20	1	1.0000	3C
**Allopatric anemone *vs.* extracts**				
Blastula 1d	25	1	1.0000	3D
Early Brachiolaria – 3d	25	1	1.0000	3D
Late Brachiolaria – 6d	25	1	1.0000	3D
**Sympatric crab *vs.* adults from João Fernandes**				
-NC – set 1	14	1	<0.0001	4A
-NC – set 2	16	1	<0.0001	4A
NC – set 1	13	1	0.0026	4A
NC – set 2	16	1	0.0001	4A
+NC	13	1	<0.0001	4A
**Allopatric crabs *vs.* adults from João Fernandes**				
-NC – set 1	12	1	0 0186	4B
-NC – set 2	13	1	0.0196	4B
NC – set 1	11	1	0.0175	4B
NC – set 2	11	1	0.0175	4B
+NC	10	1	0.0004	4B
**Sympatric crabs *vs.* adults from Itaipu**				
-NC – set 1	10	1	0.2381	4C
-NC – set 2	10	1	0.5000	4C
NC – set 1	8	1	0.5000	4C
NC – set 2	8	1	0.1000	4C
+NC	11	1	0.0005	4C
**Allopatric crabs *vs.* adults from Itaipu**				
-NC – set 1	15	1	0.0211	4D
-NC – set 2	10	1	0.0433	4D
NC – set 1	13	1	0.0196	4D
NC – set 2	9	1	0.0147	4D
+NC	9	1	0.0011	4D
